# Functional Hyaluronic Acid-Polylactic Acid/Silver Nanoparticles Core-Sheath Nanofiber Membranes for Prevention of Post-Operative Tendon Adhesion

**DOI:** 10.3390/ijms22168781

**Published:** 2021-08-16

**Authors:** Chih-Hao Chen, Yuan-Hsun Cheng, Shih-Heng Chen, Andy Deng-Chi Chuang, Jyh-Ping Chen

**Affiliations:** 1 Department of Plastic and Reconstructive Surgery, Chang Gung Memorial Hospital at Keelung, Keelung 20401, Taiwan; chchen5027@gmail.com (C.-H.C.); andy.d.chuang@gmail.com (A.D.-C.C.); 2 Department of Plastic and Reconstructive Surgery, Chang Gung Memorial Hospital at Linkou, Collage of Medicine, Chang Gung University, Kwei-San, Taoyuan 33305, Taiwan; shihheng@mac.com; 3 Department of Chemical and Materials Engineering, Chang Gung University, Kwei-San, Taoyuan 33302, Taiwan; cf30039@yahoo.com.tw; 4 Research Center for Food and Cosmetic Safety, Research Center for Chinese Herbal Medicine, Linkou Campus, College of Human Ecology, Chang Gung University of Science and Technology, Taoyuan 33305, Taiwan; 5 Department of Materials Engineering, Ming Chi University of Technology, Tai-Shan, New Taipei City 24301, Taiwan

**Keywords:** electrospinning, core-sheath nanofibers, anti-adhesion, silver nanoparticles, polylactic acid, hyaluronic acid

## Abstract

In this study, we prepared core-sheath nanofiber membranes (CSNFMs) with silver nanoparticles (Ag NPs) embedding in the polylactic acid (PLA) nanofiber sheath and hyaluronic acid (HA) in the nanofiber core. The PLA/Ag NPs sheath provides mechanical support as well as anti-bacterial and anti-inflammatory properties. The controlled release of HA from the core could exert anti-adhesion effects to promote tendon sliding while reducing fibroblast attachment. From the microfibrous structural nature of CSNFMs, they function as barrier membranes to reduce fibroblast penetration without hampering nutrient transports to prevent post-operative peritendinous adhesion. As the anti-adhesion efficacy will depend on release rate of HA from the core as well as Ag NP from the sheath, we fabricated CSNFMs of comparable fiber diameter, but with thick (Tk) or thin (Tn) sheath. Similar CSNFMs with thick (Tk^+^) and thin (Tn^+^) sheath but with embedded Ag NPs in the sheath were also prepared. The physico-chemical properties of the barrier membranes were characterized in details, together with their biological response including cell penetration, cell attachment and proliferation, and cytotoxicity. Peritendinous anti-adhesion models in rabbits were used to test the efficacy of CSNFMs as anti-adhesion barriers, from gross observation, histology, and biomechanical tests. Overall, the CSNFM with thin-sheath and Ag NPs (Tn^+^) shows antibacterial activity with low cytotoxicity, prevents fibroblast penetration, and exerts the highest efficacy in reducing fibroblast attachment in vitro. From in vivo studies, the Tn^+^ membrane also shows significant improvement in preventing peritendinous adhesions as well as anti-inflammatory efficacy, compared with Tk and Tn CSNFMs and a commercial adhesion barrier film (SurgiWrap^®^) made from PLA.

## 1. Introduction

Postoperative tissue adhesion is a universal and critical clinical challenge. The inflammatory response after tissue injury prompts the surrounding fibroblasts to repair the injured tissue, yet excessive fibrous tissues not only hamper healing, but also result in tissue adhesion in the vicinity. The probability of such a phenomenon can reach 67–100% after gastrointestinal surgery and 4–30% after tendon injury and repair [1,2,3]. In the context of tendon injury, the surrounding fibroblasts aggregate to repair the injured tissue. Nonetheless, the excessive fibroblast proliferation and protein synthesis can lead to formation of a layer of dense connective tissue over the tendon and surrounding tissues, causing tendon adhesion. This in turn restricts joint movement and results in compromised tendon gliding and pain. In such cases, complex surgical intervention is required [4]. In these cases, prevention of peritendinous adhesion is desirable with an ideal biomimetic tendon sheath surrogates to allow free and smooth movement of the encased tendon, while simultaneously maintaining a permeable barrier between the tendon and the peritendinous tissue. Such a barrier would prevent infiltration of fibroblasts and decrease the incidence of unwanted tendon adhesion during the healing process, while the permeability of the barrier would allow diffusion of cytokines, nutrients, and wastes to and from the recovering tendon. 

Polylactic acid (PLA) is a biocompatible and biodegradable polymer with widespread applications in medicine and tissue engineering [5,6,7,8]. It is one of the most promising biodegradable polymers made from renewable sources to be used in nanocomposites [9,10]. PLA has shown excellent anti-adhesion properties both in the abdominal cavity and after tendon surgery, but its extensive application is limited by its hardness, hydrophobicity, and slow degradation rate [4,11,12]. Still, by making modifications of PLA scaffolds via coating, grafting, or blending with other materials, the intrinsic properties of PLA could be tuned to broaden the applicability of PLA-based scaffolds [11,13,14,15,16,17]. For tendon repair, electrospun PLA nanofiber membranes have shown promising results as an effective tendon sheath surrogate [18,19,20]. Such a design provides a desired mechanical barrier between the injured tendon and its surrounding, while taking advantage of the non-cytotoxic nature of the material and the permeability offered by the microfibrous structure for nutrients exchange. By further modifying the fundamental polymeric nanofiber design through structural change or the coalescence of novel agents to the polymer fibers, desirable traits and improved functionality could be introduced [21,22,23].

For this purpose, modifying electrospun nanofiber structure and grafting or loading nanofibers with functional molecules can introduce different attributes into the final nanofiber design [22,24,25]. In particular, a core-sheath nanofiber structure produced via co-axial electrospinning allows fabrication of dual compartment nanofibers endowed with multi-functionality [26]. Such designs would allow controlled release of therapeutics within the core while maintaining the structural integrity of the fiber, which is supported through the outer sheath layer [27,28]. Additionally, by modulating the flow rate of spinning solutions fed to a co-axial spinneret, core-sheath nanofibers with varying core-to-sheath ratios but comparable fiber diameters may be produced with well controlled electrospinning conditions [29]. In these instances, a core-sheath nanofiber with a large core diameter and a thin sheath thickness may show faster release of the therapeutic molecules from the core, together with fast elution of embedded functional nanoparticles from the sheath to exert improved multi-functionality. 

**Scheme 1:**
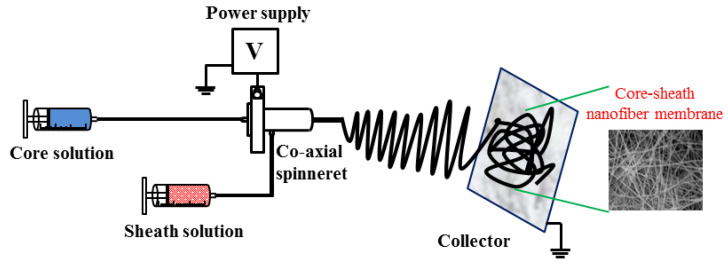
The schematic diagram for preparation of core-sheath nanofiber membranes (CSNFMs). The sheath spinning solution contains polylactic acid in methylene chloride/N,N’-dimethylformamide. For silver nanoparticles (Ag NPs)-embedded CSNFMs, the sheath spinning solution contains AgNO_3_, which is exposed to UV light beforehand to form Ag NPs. The core spinning solution contains hyaluronic acid and polyethylene oxide in formic acid.

With its good biocompatibility and biodegradability as well as nonimmunogenic properties, hyaluronic acid (HA) has been used extensively for biomedical applications [30,31]. Previously, we designed core-sheath nanofiber membranes (CSNFMs) with hyaluronic acid (HA)-infused core and silver nanoparticles (Ag NPs)-imbedded polycaprolactone (PCL) sheath, to provide controlled release of HA and Ag NPs from the membranes [24]. Such a design was shown to be effective in providing anti-adhesion, anti-bacterial, and anti-inflammatory activities via a synergetic effect of HA and Ag NPs. Despite the advantages offered through design of such CSNFMs, we postulate that the overall efficacy of an anti-adhesion membrane would depend on the core-sheath ratio of the nanofiber, since varied release rate of HA and Ag NPs may affect the collective effects from both agents. Toward this end, we used co-axial electrospinning to prepare CSNFMs with comparable fiber diameter but different core-sheath ratios (Scheme 1). Considering the polymer used in a commercial anti-adhesion film SurgiWrap^®^, we also replaced PCL in the sheath with polylactic acid (PLA) for its favorable mechanical properties and biocompatibility, to control the release of lubricating HA from the core. The Ag NPs were embedded in the PLA sheath for the anti-bacterial and anti-inflammatory activity. By modulating the release rate of HA and Ag NPs by changing the core-sheath ratio, we aim to determine the optimal design of such CSNFMs to prevent post-surgical tendon adhesion in vivo.

## 2. Results and Discussion

### 2.1. Preparation and Characterization of Core-Sheath Nanofiber Membranes (CSNFMs)

**Figure 1:**
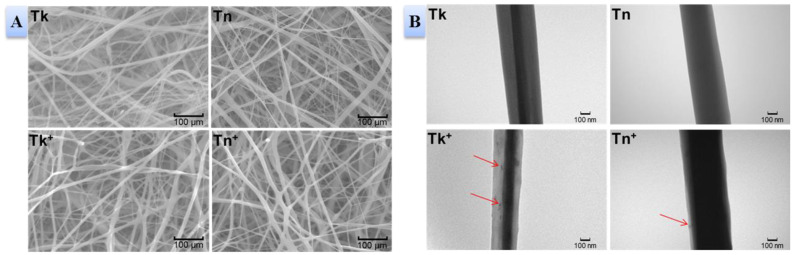
The scanning electron microscopy (SEM) (**A**, bar = 100 μm) and transmission electron microcopy (TEM) (**B**, bar = 100 nm) images of Tk, Tn, Tk^+^, and Tn^+^ core-sheath nanofiber membranes (CSNFMs). The red arrows in (**B**) indicate Ag NPs in the sheath.

We determined the morphology and the diameter of nanofibers by scanning electron microscopy (SEM). From Figure 1A, we can observe the four types of CSNFMs fabricated in this study, thick-sheath (Tk) and thin-sheath (Tn) without Ag NPs and thick-sheath (Tk^+^) and thin-sheath (Tn^+^) with Ag NPs, to comprise continuous and beadless nanofibers from co-axial electrospinning. Measurements by ImageJ software indicate the fiber diameter is 643 ± 119 nm for Tk, 680 ± 167 nm for Tn, 692 ± 165 nm for Tk^+^, and 725 ± 226 nm for Tn^+^, with no significant difference found among all membranes. The mean pore flow pore diameters measured by capillary flow porometry are 1.89 ± 0.40, 2.28 ± 0.33, 1.80 ± 0.62, and 1.94 ± 0.62 μm for Tk, Tn, Tk^+^, and Tn^+^, respectively. As with fiber diameter, there is no significant difference in pores size among all membranes, although all are associated with a pore size small enough to prevent fibroblast penetration. The transmission electron microcopy (TEM) images of the nanofibers show a distinct core-sheath structure (Figure 1B). Differences in nanofiber structure with thick sheath (Tk and Tk^+^) or thin sheath (Tn and Tn^+^) is clearly observed, depending on individual flow rates of the polymer solutions in the co-axial spinneret. Both spherical and rod-like Ag NPs with size ranging from 10 nm to 40 nm are observed to be embedded in the sheath of Tn^+^ and Tk^+^ CSNFMs after reducing the AgNO_3_-containing sheath spinning solution with 254 nm UV light. 

**Figure 2:**
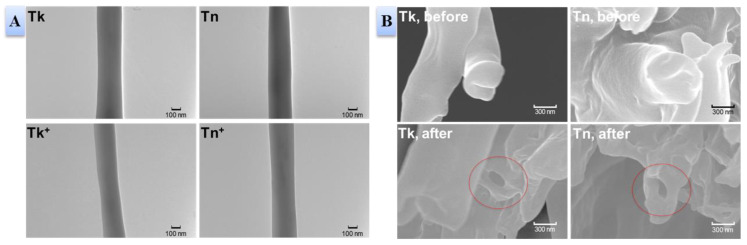
(**A**) The transmission electron microcopy (TEM) images of Tk, Tn, Tk^+^, and Tn^+^ CSNFMs after immersion in PBS for two weeks (bar = 100 nm). (**B**) The field-emission scanning electron microscopy (FE-SEM) images of Tk and Tn core-sheath nanofiber membranes (CSNFMs) before and after immersion in PBS for two weeks (bar = 300 nm). The red circles indicate the hollow core structure after eluting HA from the core.

The core-sheath structure was further demonstrated from TEM images after immersing the CSNFMs in phosphate buffered saline (PBS) for 14 days to release HA and Ag NPs. As shown in Figure 2A, there is diminished color within the core area after eluting HA form the core, together with disappearance of dark spots (Ag NPs) for Tk^+^ and Tn^+^ after the release of Ag NPs from the sheath. Further proof of the core-sheath structure was provided by comparing the field-emission scanning electron microscopy (FE-SEM) images of the cross sections of the Tk and Tn nanofibers, before and after immersion in PBS. After losing HA from the core in PBS for 14 days, a clear hollow core is evident for both CSNFMs regardless of sheath thickness, supporting successful fabrication of CSNFMs (Figure 2B). 

**Figure 3:**
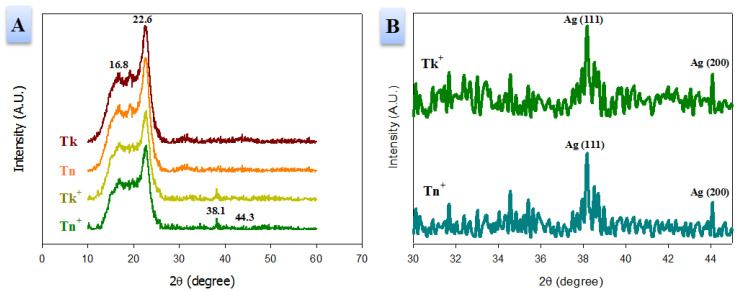
The X-ray diffraction (XRD) analysis of Tk, Tn, Tk^+^, and Tn^+^ CSNFMs (**A**) and high resolution XRD spectra of Tk^+^ and Tn^+^ CSNFMs (**B**).

From X-ray diffraction (XRD) analysis, all CSNFMs display two broad peaks at 2θ = 16.8° and 22.6° due to PLA (Figure 3A). A similar peak intensity for Tk (Tn) with Tk^+^ (Tn^+^) indicates Ag NPs do not influence the crystallinity of PLA. Furthermore, the high-resolution XRD spectra of Tk^+^ and Tn^+^ in Figure 3B clearly display two additional Ag peaks at 2θ = 38.1° and 44.3°, which correspond to the (111) and (200) crystalline planes of Ag. This confirms the formation of Ag NPs after UV-reduction of AgNO_3_ in PLA solution prepared from methylene chloride/dimethylformamide (MC/DMF) [32]. This also coincides with JCPDS file No. 04-0783 for the face-centered cubic structure of Ag NPs [33]. 

**Figure 4:**
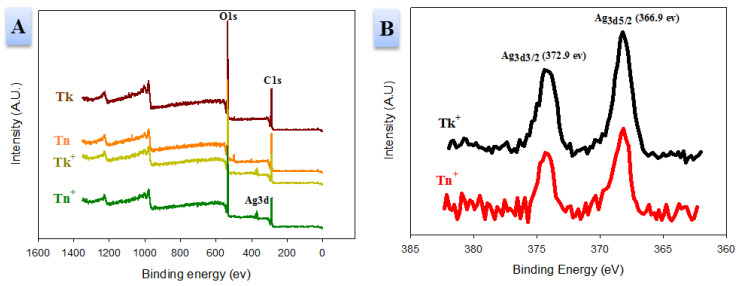
The X-ray photoelectron spectroscopy (XPS) analysis of Tk, Tn, Tk^+^, and Tn^+^ CSNFMs (**A**) and high resolution XPS spectra of Tk^+^, and Tn^+^ CSNFMs (**B**).

From X-ray photoelectron spectroscopy (XPS) analysis, characteristic peaks assigned to Ag only appear for TK^+^ and Tn^+^ (Figure 4A), indicating some Ag NPs are exposed on the surface of those nanofibers since the detection limit of XPS is only restricted to several nanometers form the surface. We could further confirm the existence of Ag NPs on the nanofiber surface from the high-resolution XPS spectra in Figure 4B, which shows Ag peaks at 366.9 eV (Ag_3d3/2_) and 372.9 eV (Ag_3d5/2_) for Tk^+^ and Tn^+^ (Figure 4B). The elemental compositions of different CSNFMs from XPS analysis are shown in Table 1. The approximate amounts of Ag NPs on fiber surface are estimated to be 0.03% and 0.01% for Tk^+^ and Tn^+^, respectively. This low value is expected, as only few Ag NPs will be present on nanofiber surface with the electrostatic repulsion force generated from the positive voltage during electrospinning [34]. 

### 2.2. Protein Permeability, Release Property and Anti-Bacterial Activity

For an anti-adhesion barrier membrane to be effective, the fibroblast penetration and migration should be prevented without impeding the permeation of nutrients that are important during tendon healing. To confirm the permeation properties, the permeation coefficient of a sample nutrient (bovine serum albumin, BSA) thorough the CSNFMs was determined at 37 °C in a side-by-side permeation cell and compared with that of a commercial anti-adhesion barrier film (SurgiWrap^®^). As shown in Table 2, there is no significant difference in permeability coefficient of BSA through all CSNFMs while the permeability coefficient of BSA was almost three orders lower in SurgiWrap^®^, reflecting the impermeable nature of SurgiWrap^®^ to BSA. Indeed, the impermeability of SurgiWrap^®^ could be related to its non-porous and dense membrane nature, which is expected to profoundly hamper nutrient transport across the barrier membrane. Despite that the same polymer (70:30 poly(L-lactide-*co*-D,L-lactide)) was used in SurgiWrap^®^ and in the sheath of CSNFMs, the use of electrospinning to produce macroporous CSNFMs could drastically improve the membrane permeability of BSA. This suggests that despite that CSNFMs are associated with small pore size (~2 µm), they do not hamper the transport of nutrients through the barrier during tendon healing, announcing the advantage of using such macroporous membranes for anti-adhesion purposes over a dense polymer film such as SurgiWrap^®^ [35]. 

**Figure 5:**
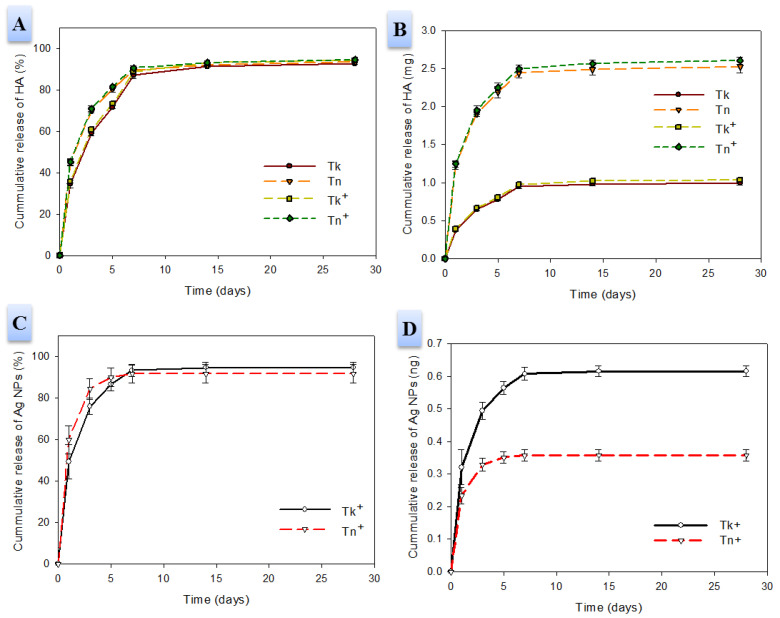
The cumulative release percentage (**A**) and cumulative released mass (**B**) of HA. The cumulative release percentage (**C**) and cumulative released mass (**D**) of Ag NPs.

The individual release profiles of HA and Ag NPs were studied next. During the first week, all CSNFMs showed rapid and steady HA release, to reach ~90% cumulative HA release percentage (calculated based on HA used for preparation of the CSNFMs) after 10 days (Figure 5A). Nonetheless, CSNFMs with thin sheath (Tn and Tn^+^) shows higher HA release rate than their thick-sheath counterparts (Tk and Tk^+^), as less diffusion length is required for diffusion of HA through the sheath. Considering the cumulative mass of HA released, which is directly related to its anti-adhesion functionality, it is similar for Tn and Tn^+^, but ~3 times higher than that of Tk and Tk^+^, with higher loading of HA in the large core of thin-sheath nanofibers (Figure 5B). For release of Ag NPs, the quantity of embedded nanoparticles in the membranes was determined beforehand. From the amount of silver nitrate used for preparation of CSNFMs, the theoretical contents of Ag NPs are 0.76 and 0.49 mg/g membrane in Tk^+^ and Tn^+^, respectively, assuming 100% reduction efficiency of Ag^+^ to Ag NPs by photo-reduction of Ag^+^ ions with UV light in MC/DMF. The actual amount Ag NPs embedded in CSNFMs from coupled plasma optical emission spectrometry (ICP-OES) analysis revealed 0.65 and 0.39 mg/g membrane for Tk^+^ and Tn^+^, respectively. This high reduction efficiency (>80%) can facially produce dispersed Ag NPs in PLA polymer solution to be used as the sheath spinning solution. Indeed, a previous study confirmed the use of DMF to reduce Ag^+^ to Ag in a mixed solvent system, where Ag NPs could be formed in situ without agglomeration during crystal growth for embedding in a polymer matrix [36]. Using the amount of Ag NPs loaded in Tn^+^ and TK^+^, the cumulative release percentage profiles of Ag NPs is similar for both CSNFMs, which is faster than HA and reached ~90% within five days (Figure 5C). Although the release percentage is similar, the released mass of Ag NPs for Tk^+^ is about two times that of Tn^+^, as more Ag NPs are embedded in Tk^+^ with its thick sheath (Figure 5D). In an aqueous environment, Ag NPs on the surface of nanofibers are readily to react with water and mostly released into the solution in form of silver ions. After the initial release of surface Ag, the release process becomes diffusion limited. However, with the large surface area of nanofibers, it is difficult to have extended release of Ag. Since an inflammatory response after tendon surgery is expected to be more severe during the first few days, the fast release of Ag NPs from CSNFMs is deemed suitable to induce early anti-inflammatory and subsequent anti-adhesion effects during tendon healing [37]. 

**Figure 6:**
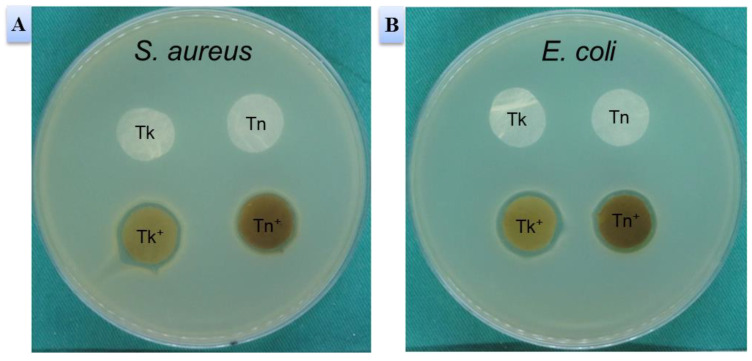
The antibacterial activity of CSNFMs determined from the bacteriostatic rings for *S. aureus* (**A**) and *E. coli* (**B**). A disk shaped membrane with 1.5 cm diameter is used in the study and the weight of Ag NPs in Tn^+^ and TK^+^ are ~0.0195 and ~0.0325 mg, respectively.

For anti-bacteria activity of CSNFMs, we used two typical bacteria strains causing infection to determine the bacteriostatic efficacy against *S. aureus* and *E. coli* with released Ag NPs. As shown in Figure 6, bacteriostatic rings were readily observable around Tk^+^ and Tn^+^ membrane disks for both bacteria, while no zone of inhibition was observed for Tk and Tn. With diffusion of Ag NPs from the pre-wetted membrane to the surrounding test area on an agar plate, only Ag NPs-embedded CSNFMs can display anti-bacterial activity, by incorporating released Ag into the cell membrane and inducing cell death [38]. The corresponding zone of inhibition of Tk^+^ is 1.29 ± 0.11 cm^2^ for *E. coli* and 0.97 ± 0.15 cm^2^ for *S. aureus* with difference in their wall structure. The *S. aureus* is a Gram-positive strain, which has thicker peptidoglycan layer in the cell wall than the Gram-negative *E. coli*, making the penetration of Ag NPs more difficult when interfering with the metabolic pathways [39]. In comparison, the inhibition zone of Tn^+^ is 1.04 ± 0.14 cm^2^ for *E. coli* and 0.92 ± 0.14 cm^2^ for *S. aureus* as less Ag NPs were released from the membranes (Figure 5D). 

### 2.3. In Vitro Cell Culture

**Figure 7:**
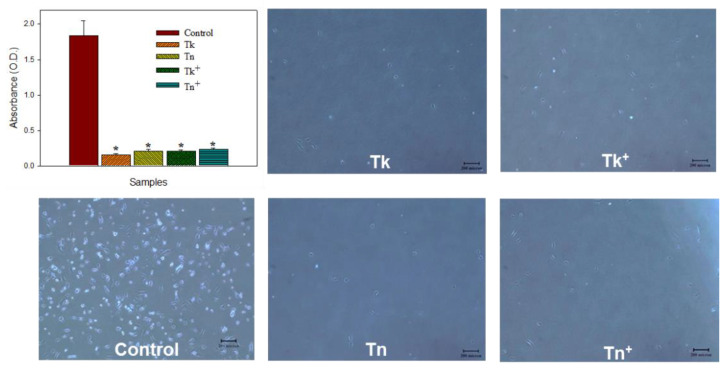
The penetration of 3T3 fibroblasts through a CSNFM in 24 h by determining penetrated cell number from solution absorbance with MTS assays, and direct microscopic observation of penetrated cells (bar = 200 μm). The control is without a CSNFM. * *p* < 0.05 compared with control.

The penetration of 3T3 fibroblasts during in vitro cell culture was tested by serum concentration-induced cell migration to the lower chamber of a double chamber dish, by placing a CSNFM at the bottom of a cell insert fitted in the upper chamber. The control is without using a membrane in the cell culture insert. The amount of migrated 3T3 fibroblasts to the lower chamber was determined from the MTS assays. As shown in Figure 7, the penetrated cells in all CSNFMs were only ~10% that in the control group, with no significant difference found between different membranes. Microscopic observation of the bottom chamber surface also confirmed these findings, with minimal cells found on the bottom chamber surface using a CSNFM as a barrier, in contrast to abundant cells found in the control. These findings are consistent with the results from pore size analysis, as the pore size of all CSNFMs was much smaller than the size of fibroblasts to effectively block cell penetration. Taken together, the hindered cell migration though a CSNFM supports the choice of such a macroporous structured membrane to block fibroblast penetration, mitigate tendon adhesion, and ultimately achieve desirable anti-adhesion effects in vivo, pending evaluation of their effects on cell attachment and cytotoxicity. 

**Figure 8:**
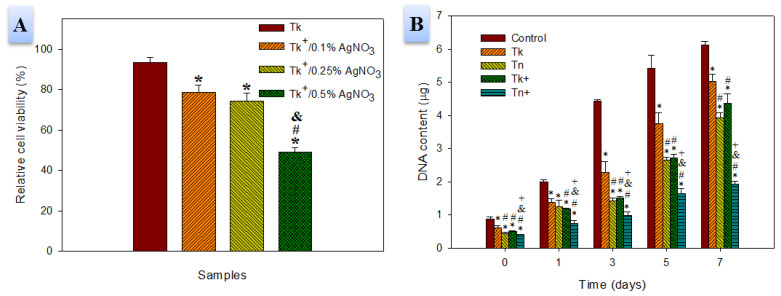
(**A**) The cytotoxicity tests of CSNFMs using the indirect contact method. * *p* < 0.05 compared with Tk; ^#^ *p* < 0.05 compared with Tk^+^/0.1% AgNO_3_; ^&^ *p* < 0.05 compared with Tk^+^/0.25% AgNO_3_. (**B**) The attachment and proliferation of 3T3 fibroblasts on control (TCPS) and CSNFMs from DNA assays. * *p* < 0.05 compared with control; ^#^ *p* < 0.05 compared with Tk; ^&^ *p* < 0.05 compared with Tn; ^+^ *p* < 0.05 compared with Tk^+^.

The possible cytotoxicity of Tk and Tk^+^ was determined using the indirect method by culturing fibroblasts with a 24-h extract of a CSNFM membrane, to realize whether any of the constituents of CSNFMs have cytotoxic effects. By normalizing the MTS absorbance of the sample (extract) with that of the control (fresh cell culture medium), the relative cell viability was 93.3 ± 2.7% for Tk, indicating no cytotoxicity is associated with all constituents within this CSNFM, excluding Ag NPs. (Figure 8A). The relative cell viability decreased to 78.8 ± 3.4% and 74.5 ± 3.8% for Tk^+^ prepared with 0.1% and 0.25% AgNO_3_, with no significant difference. Both samples also meet the requirement for a non-toxic medical device per ISO 10993-5 standard (>70% viability). However, for Tk^+^ membranes prepared with 0.5% AgNO_3_, cell viability drastically decreased to 49.0 ± 2.3%, implicating overdosing of AgNO_3_ in the shell spinning solution could raise the cytotoxicity of CSNFMs. This may arise due to incomplete reduction of Ag^+^, with cytotoxicity induced by Ag NPs to be much lower than that caused by Ag^+^ on mammalian cells [40]. Taken together, we confirm a 0.25% AgNO_3_ dose in the sheath spinning solution would be ideal, considering both bacteriostatic effect and tolerable cytotoxicity, which was adopted for preparation of Tn^+^ and Tk^+^ CSNFMs.

The attachment and proliferation of fibroblasts was studied by seeding 3T3 cells on CSNFMs and cultured for seven days, before determining cell numbers with DNA assays. The cell attachment on all CSNFMs was much lower than that on a tissue culture polystyrene (TCPS) plate that is used as a control (Figure 8B), suggesting that all membranes developed in this study could reduce fibroblast attachment. Nonetheless, the Tn^+^ shows significant reduction of adhered cell number from other membranes. The drastic drop in number of adhered cells in CSNFMs compared with the control is consistent with our previous studies and may be due to the synergistic effects of HA and Ag NPs as Tk^+^ (Tn^+^) showed significant decrease of attached fibroblasts compared with Tk (Tn) [41]. Previously, HA was shown to have a strong influence on the adhesion of fetal fibroblasts [42], while Ag NPs are known to reduce fibroblast adhesion [43]. The membranes with thinner sheath (Tn and Tn^+^) also demonstrated lower cell attachment than Tk and Tk^+^, indicating more HA released from thin-sheath membrane is beneficial in reducing cell attachment from combined lubrication and anti-adhesion effects [44]. 

**Figure 9:**
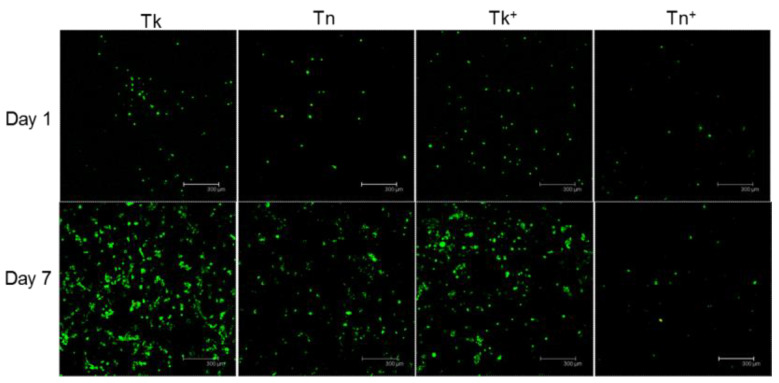
The Live/Dead staining of fibroblasts on CSNFMs by calcein AM and propidium iodide for live (green) and dead (red) cells after cultured for one and seven days and observed by confocal laser scanning microscopy. Bar = 300 μm.

For cell proliferation, the cell number on day 1, 3, 5, and 7 followed the same trend as day 0, with TCPS showing the maximum cell number and Tn^+^ the minimum (Figure 8B). We can ascribe this effect on cell proliferation to released HA and Ag NPs, where previous reports found hindrance of fibroblast proliferation caused by high concentrations of HA [45,46], and reduction in fibroblasts proliferation reported for Ag NPs [47]. The Live/Dead staining images of fibroblasts on CSNFMs in Figure 9 support the trend observed in Figure 8B with more cells observed on Tk and Tk^+^ relative to that on Tn on day 7, but the least number of viable cells were found on Tn^+^.

**Figure 10:**
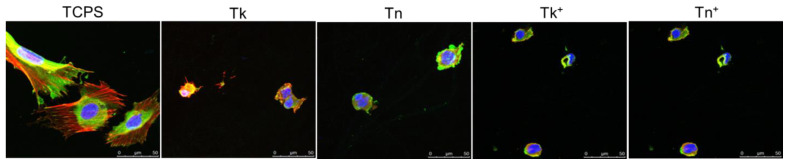
The cytoskeletal F-actin (red) distribution and vinculin focal adhesion protein (green) expression of fibroblasts after cultured on control (TCPS) and different CSNFMs for one day and examined under a confocal laser scanning microscope. The cell nuclei were counterstained with DAPI (blue). Bar = 50 μm.

To elucidate the molecular mechanism of reduced fibroblast attachment on the membrane, the expression of a focal adhesion protein (vinculin) as well as cytoskeletal actin distribution in 3T3 fibroblasts were observed under a confocal microscope after cultured for 24 h. As shown in Figure 10, fibroblasts attached to TCPS showed distinctive cellular spreading with flattened cell morphology to result in enhanced vinculin (green fluorescence) expression and a well-distributed F-actin fibrous cytoskeleton (red fluorescence). By comparison, the fibroblasts found on CSNFMs were relatively round, showed minimal vinculin expression, and showed restricted cytoskeletal distribution without obvious stretching and cellular spreading, indicative of poor cell adhesion [48]. This may be supported from a previous report reporting the negative impact of HA on cellular adhesion and migration of skin dermal fibroblasts [49]. A synergistic effect was found by combining Ag NPs with HA as Tn^+^, with Ag NPs embedded in the thin sheath and the highest amount of released HA, which demonstrated the least staining of vinculin by inhibiting cell adhesion as well as suppressing cell spreading. Thus, we used Tn^+^ membrane to compare with Tn in the animal study to elucidate the effects from Ag NPs in vivo. 

### 2.4. In Vivo Studies

**Figure 11:**
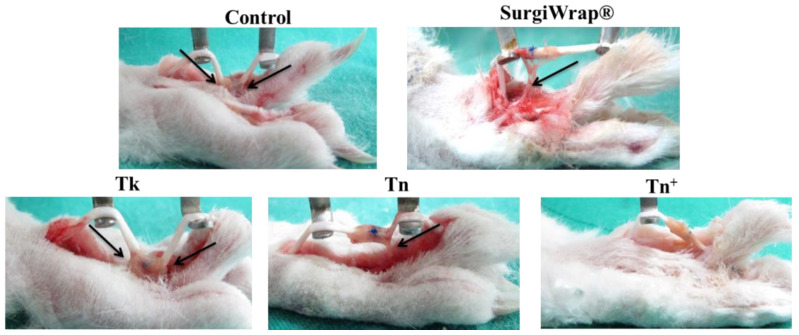
The rabbit flexor tendons observed three weeks post-operatively. Note the substantial peritendinous adhesions (black arrows) in the control and Tk groups, with fewer adhesions observed in the SurgiWrap^®^ and Tn groups. Peritendinous adhesions are absent in the Tn^+^ group.

Using SurgiWrap^®^ as a positive control, Tn vs Tk for the effect of HA, and Tn vs Tn^+^ for the effect of Ag NPs, we carried out animal studies with a flexor digitorum profundus (FDP) tendon rupture model in rabbits. Direct visualization of the rabbit flexor tendons showed similar extents of peritendinous adhesions for the control (untreated) group and the Tk group, with the flexor tendon being firmly attached to the surrounding tissue with tight adhesions (Figure 11). These adhesions are composed of excessive fibrous tissues in which fibroblast aggregated in the process of wound repair. The adhesions also completely covered the injured tendon such that a strong force was required to separate the tendon from the surrounding tissues. In contrast to the control and Tk groups, mild degree of peritendinous adhesions with minimal adhesion bands were observed in the positive control (SurgiWrap^®^) and Tn groups, while the Tn^+^ group showed a nearly complete lack of adhesion (Figure 11). 

**Figure 12:**
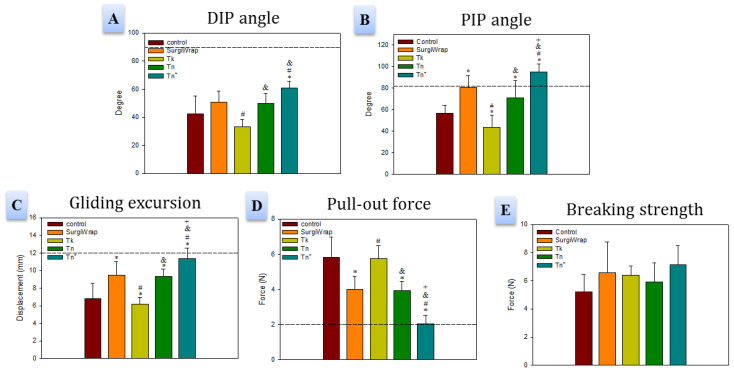
The assessment of peritendinous adhesions three weeks post-operation for distal interphalangeal (DIP) flexion angle (**A**), proximal interphalangeal (PIP) flexion angle (**B**), tendon gliding distance (**C**), and pull-out force (**D**). The dotted line represents the average value found for normal flexor digitorum profundus tendons. (**E**) Comparison of the breaking strength of healed tendons at three weeks. * *p* < 0.05 compared with control; ^#^ *p* < 0.05 compared with SurgiWrap^®^; ^&^ *p* < 0.05 compared with Tk; ^+^ *p* < 0.05 compared with Tn. The data are expressed as mean ± standard deviation (*n* = 8).

From biomechanical analysis, the flexion angles of the distal interphalangeal (DIP) and proximal interphalangeal (PIP) joints show a comparable pattern to the gross examination of peritendinous adhesions, with the untreated control group performing similarly to the Tk group (Figure 12A,B). Though there are no significant differences between these two groups for the DIP joint flexion angles, the PIP joint flexion angles of the Tk group were significantly worse than the untreated control group. Findings for the SurgiWrap^®^ and Tn groups were also alike without any significant differences between them but showed improvement over control and Tk. Most importantly, the Tn^+^ group performed significantly better than all other groups. A similar pattern is also observed for tendon gliding excursion and pull-out force (Figure 12C,D). These findings are directly compatible with the amount of peritendinous adhesions observed from gross examination. Mechanical testing for the force tolerated before rupture of healed tendon revealed no significant difference among all groups (Figure 12E). This supports the idea that neither CSNFMs or SurgiWrap^®^ will affect the normal process of tendon healing [50]. Based on these findings, wrapping the injured tendon with a Tn^+^ CSNFM is deemed the most effective treatment to exert the anti-adhesion effect while still allowing normal healing of the contained tendon. 

**Figure 13:**
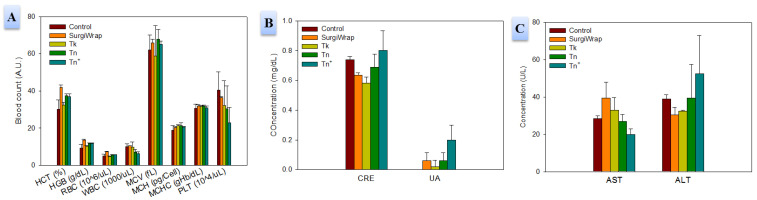
The blood testing results at one week postoperatively from complete blood count (**A**), renal (**B**), and liver (**C**) functions assessments. HCT, hematocrit; HGB, hemoglobin; RBC, red blood cell; WBC, white blood cell; MCV, mean corpuscular volume; MCH, mean corpuscular hemoglobin; MCHC: mean corpuscular hemoglobin concentration; PLT, platelet; CRE, creatinine; UA, uric acid; AST, aspartate aminotransferase; ALT, alanine aminotransferase. The data are expressed as mean ± standard deviation (*n* = 8).

Considering adverse effects from treatments, the blood samples taken from the animals one week post-operatively revealed no significant differences between the control and all experiment groups from complete blood count (Figure 13A), renal (Figure 13B), and liver (Figure 13C) function assessments. The results verify that wrapping CSNFMs around the injured tendons of the rabbits will not cause noticeable inflammatory responses or metabolic abnormalities in the liver and kidney. Hence, the materials did not directly influence the rabbits’ physiological conditions. 

**Figure 14:**
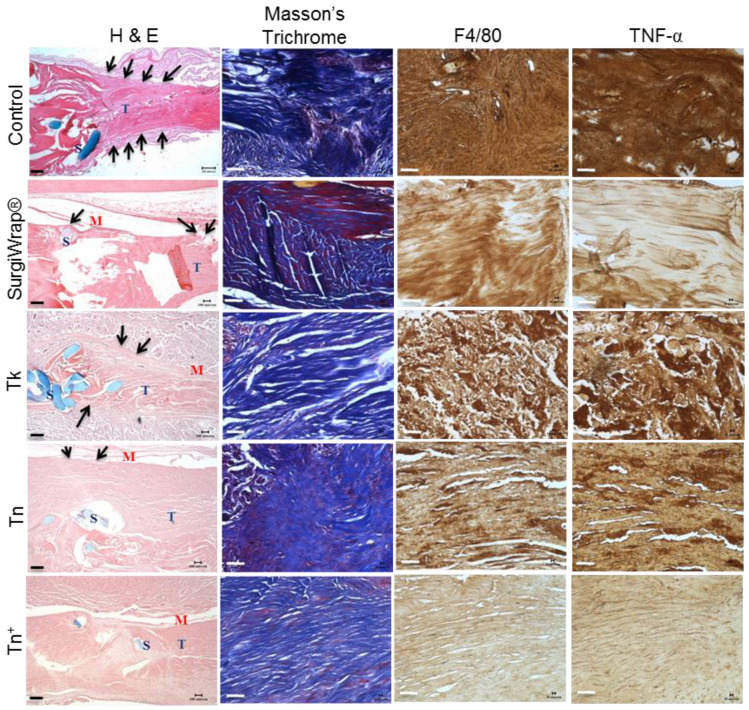
The histological analysis of untreated tendons (control group), tendons treated with SurgiWrap^®^, and tendons treated with Tk, Tn, or Tn^+^ CSNFMs. The hematoxylin-eosin (H&E) staining shows adhesion tissues around repaired tendon (black arrows) as well the position of suture (S), tendon (T), and membrane (M) (bar = 200 μm). The Masson’s trichrome stain and immunohistochemical (IHC) staining of TNF-α and F4/80 are included (bar = 50 μm).

From histological examination in Figure 14, the hematoxylin and eosin (H&E) stain of the tendon tissue sections revealed severe adhesions between the tendon and the surrounding tissues for the unwrapped control group, leading to the severe restriction of tendon movement. The Tk membrane treated tendon also exhibited postoperative adhesion between the tendon suture and its surrounding tissues. In the SurgiWrap^®^ and Tn groups, loose fibrous tissues formed around the repaired tendon, resulting in mild adhesion. There is a noticeable gap between the membrane and the surrounding tissue, allowing better tendon sliding. Compared with other groups, the Tn^+^ group exhibited excellent anti-adhesion effect with the least loose fibrous tissue formation while still retained a sizable interval between the tendon and its surrounding tissue. Masson trichrome staining was used to observe collagen fiber distribution and morphology (Figure 14). In the unwrapped control group, collagen arrangement was relatively irregular. In contrast, the Tn^+^ CSNFM group demonstrated the most regular collagen arrangement.

When a tendon is injured or damaged during surgery, the body initiates an inflammatory response in response to the insult with macrophages aggregate in the wound, attaching to the damaged tissue. As a glycoprotein member of the epidermal growth factor-transmembrane 7 family, F4/80 is expressed on the majority of tissue macrophages as a widely used murine macrophage marker [51]. F4/80 immunohistochemical (IHC) staining can, thus, be used to determine the extent of postoperative tendon inflammation [52]. The F4/80 IHC staining image for the untreated control group exhibited deep staining (Figure 14), implying the abundance of F4/80 expression in the tendon arising from a more severe inflammatory response. The SurgiWrap^®^, Tk, and Tn groups also had marked staining for F4/80, though on a less severe level than the untreated control. Compared to other groups, the Tn^+^ group exhibited considerably lighter staining, indicating a milder inflammation response. Because Ag NPs exerted an anti-inflammatory effect, postoperative inflammation was milder in the Tn^+^-wrapped group [53]. Tumor necrosis factor-α (TNF-α) is a major pro-inflammatory cytokine that is expressed in the early stage of cell inflammation and induces apoptosis [54]. As TNF-α is produced by macrophages/monocytes during acute inflammation, we also used IHC staining to investigate the presence of this inflammatory cytokine around the site of repaired tendon. The staining intensity of the pro-inflammatory cytokine TNF-α in Tn^+^ is much weaker than all the other groups, with light brown color, endorsing the anti-inflammation performance of this CSNFM due to Ag NPs (Figure 14). This effect is consistent with a recent study confirming the anti-inflammatory activity of Ag NPs originating from inhibition of production of pro-inflammatory cytokines (IL-1β, IL-6 and TNF-α) in macrophages [55]. Using both TNF-α and F4/80 as macrophage markers, we confirm from IHC staining the dominance of the Ag NPs-embedded CSNFM (Tn^+^) over the commercially available barrier material (SurgiWrap^®^) as well as CSNFMs without Ag NPs (Tk and Tn) in preventing post-surgical tendon inflammation in vivo. 

## 3. Materials and Methods

### 3.1. Materials 

Poly(L-lactide-*co*-D,L-lactide) (Resomer^®^ LR704S, L-Lactide:D,L-Lactide = 70:30, intrinsic viscosity = 2.4 dL/g), polyethylene oxide (PEO, molecular weight = 2,000,000 Da), silver nitrate (AgNO_3_), Dulbecco’s modified Eagle’s medium (DMEM), and actin cytoskeleton/focal adhesion staining Kit (FAK100) were purchased from Sigma-Aldrich (St. Louis, MO, USA). Hyaluronic acid (HA, average molecular weight = 1,300,000 Da) was obtained from Shandong Freda Biochem Co., Ltd. (Jinan, China). Antibiotics, LIVE/DEAD^®^ Viability/Cytotoxicity kit for mammalian cells, fetal bovine serum (FBS), and trypsin-EDTA were acquired from Life Technologies (Carlsbad, CA, USA). SurgiWrap^®^ was purchased from MAST Biosurgery (San Diego, CA, USA). CellTiter 96^®^ AQueous One Solution Cell Proliferation Assay was obtained from Promega (Madison, WI, USA).

### 3.2. Preparation of Core-Sheath Nanofibrous Membrane (CSNFMs) 

The CSNFMs were produced by co-axial electrospinning with two polymer solutions prepared separately for the core and sheath compartments. The sheath spinning solution was prepared by dissolving PLA in a 7:3 (*v*/*v*) methylene chloride (MC) and N,N’-dimethylformamide (DMF) solution to reach 5% (*w*/*v*) polymer concentration. For Ag NPs-embedded CSNFMs, the spinning solution contains 0.25% (*w*/*v*) AgNO_3_ prepared in 7:3 (*v*/*v*) MC/DMF, which was exposed to 254 nm UV light exposure at 0.1 J/cm^2^ for 3 h in a UV Crosslinker to form Ag NPs. For the core spinning solution, 0.2 g of HA and 0.05 g of PEO were dissolved in 10 mL of formic acid. A commercial electrospinning system (Falco Tech Enterprise, Co. Ltd., New Taipei City, Taiwan) was used, containing two syringe pumps connected to a high-voltage power supply and a co-axial spinneret for electrospinning. The solution flow rates for the core and sheath are 0.5 mL/h and 1 mL/h, respectively, for the CSNFMs with thick sheath (Tk) while they are 1 mL/h and 0.5 mL/h for the CSNFMs with thin sheath (Tn). The applied voltage was 25 kV and nanofibers were collected on an aluminum foil placed 15 cm from the needle tip. For Ag NPs-embedded nanofibers, termed Tk^+^ for the thick-sheath and Tn^+^ for the thin-sheath, the CSNFMs were prepared following the same fabrication condition using the Ag NPs-containing sheath solution.

### 3.3. Characterization of Core-Sheath Nanofibrous Membrane (CSNFMs) 

The morphology of nanofibers was observed under a scanning electron microscope (SEM, Hitachi S3000N, Tokyo, Japan) at 15 kV. The average fiber diameter was estimated from 100 randomly chosen nanofibers from five images (20 each) using the ImageJ software. A transmission electron microscope (TEM, Hitachi H-7500, Tokyo, Japan) was used to examine the core-sheath structure at 75 kV. The membrane pore size was measured by using a capillary flow porometer (CFRP-1500AE, Porous Materials Inc., Ithaca, NY, USA ) using Galwick^®^ with 15.9 dyne/cm surface tension. For X-ray diffraction (XRD) analysis, a Siemens D5005 X-ray diffractometer was used for scanning from 2θ = 10° to 60° at 1.2°/min scanning speed. For surface chemical composition analysis, a PHI 1600 ESCA spectrometer from Physical Electronics (Chanhassen, MN, USA) was used for high-resolution X-ray photoelectron spectroscopy (XPS). The amount of Ag NPs in the membranes was determined from coupled plasma optical emission spectrometry (ICP-OES) analysis (Agilent 725, Santa Clara, CA, USA). A side-by-side permeation chamber was to determine the diffusion of bovine serum albumin (BSA) through a membrane at 37 °C. By placing a CSNFM or SurgiWrap^®^ firmly between the two half-cells, the permeability coefficient of BSA was calculated from the time-lapsed protein concentration change in the receptor cell [56]. 

### 3.4. Release of HA and Ag NPs 

The release of HA and Ag NPs from a 1.5-cm disk-shaped CSNFM was determined in 3 mL of PBS (pH 7.4) placed in a 20 mL glass vial at 37 °C. At predetermined times, all PBS was removed and replenished with equal volume of PBS to continue the experiment. The concentration of HA in the PBS was analyzed with an ELISA kit for HA and the amount of Ag released was analyzed using ICP-OES after digesting with 5% nitric acid. The cumulative weight of HA or Ag released form the membrane was determined by adding the total amount of HA or Ag released up to a certain time. The cumulative percentage HA or Ag released form the membrane was calculated by diving the cumulative released weight with the weight initially loaded in the membrane. After the release experiments, the membranes were immersed in liquid nitrogen and broken to expose the cross section. Samples were examined for core-sheath morphology using FE-SEM (JSM-7500F, JEOL, Tokyo, Japan) and TEM (JEM-1230, JEOL, Tokyo, Japan).

### 3.5. In Vitro Cell Culture 

#### 3.5.1. Anti-Bacterial Activity

The anti-bacterial activity CSNFMs was tested against Gram-positive (*Staphylococcus aureus*, BCRC 10451) and Gram-negative (*Escherichia coli*, BCRC 11634) bacteria. The strains were cultured in 10 mL of Luria-Bertani broth for inoculation on an agar plate. Disk-shaped CSNFMs (1.5-cm diameter) were pre-wetted with PBS and placed in the agar plate for incubation at 37 °C for 24 h. The zone of inhibition was calculated from the area (cm^2^) of the bacteriostatic ring around the membrane. 

#### 3.5.2. Cell Penetration 

For cell penetration tests, a CSNFM was fitted to the bottom porous membrane of a cell insert (Transwell^®^ cell culture inserts, Corning, Tewksbury, MA, USA), which is fitted in the upper chamber of a double-chamber dish. A cell insert without a CSNFM was used as the control. NIH/3T3 mouse embryonic fibroblasts (ATCC-CRL1658) (2.5 × 10^5^ cells) diluted in DMEM/2% FBS were added to the upper chamber and the lower chamber was filled with DMEM/10% FBS. After incubated for 24 h at 37 °C, cell penetration driven by FBS concentration gradient was determined form the number of viable cells in the lower chamber by using MTS assays (CellTiter 96^®^ Aqueous Solution Cell Proliferation Assay) at 492 nm with an ELISA plate reader. The direct microscopic observation of cells in the lower chamber was done using an inverted microscope (Olympus IX-71, Tokyo, Japan).

#### 3.5.3. Cytotoxicity 

The indirect contact method (ISO 10993-5:2009, biological evaluation of medical devices– Part 5: tests for in vitro cytotoxicity) was employed to test for in vitro cytotoxicity of CSNFMs. We cultured 3T3 fibroblasts (1.0 × 10^4^ cells) at 37 °C with the extraction medium of membranes, which was prepared by incubating 15-mm diameter CSNFMs in 90% DMEM/10% FBS at 37 °C for 24 h. The MTS assay was used to measure the solution absorbance values at 492 nm and normalized to that from cells cultured with 90% DMEM/10% FBS.

#### 3.5.4. Cell Attachment and Proliferation 

We seeded 3T3 fibroblasts on a pre-wetted disc-shaped CSNFM of 15 mm diameter at 1.0 × 10^4^ seeding density and carried out cell culture at 37 °C with DMEM supplemented with 10% (*v*/*v*) FBS and 1% (*v*/*v*) antibiotic-antimycotic. A control was prepared by seeding the same amount of fibroblasts on tissue culture polystyrene (TCPS) plates. After 3 h, the membranes or TCPS plates were washed with DMEM to remove loosely attached cells for determination of cell attachment efficiency at day 0. The cell culture continued for up to seven days with medium change every other day. The cell proliferation rate was determined from number of cells attached to the CSNFMs (or TCPS plates) on day 1, 3, 5, and 7. The cell number was determined from the DNA content with Hoechst 33258 DNA assays. The distribution of live and dead fibroblasts on CSNFMs were assessed on day 1 and 7 using a LIVE/DEAD^®^ Viability/Cytotoxicity kit containing the fluorescent molecular probe calcein AM and propidium iodide to visualize live cells (in green) and dead cells (in red) under a confocal laser scanning microscope (Zeiss LSM 510 Meta, Oberkochen, Germany).

#### 3.5.5. Cytoskeletal and Vinculin Staining 

Triple fluorescence staining of the cytoskeleton, vinculin, and nucleus were done for 3T3 cells cultured on the CSNFMs for 24 h. The membranes were washed with PBS and treated with 4% paraformaldehyde for 20 min; followed by permeabilizing cells with 0.1% Triton X-100 for 10 min. The cytoskeletal distribution was examined by incubating cultured cells with tetramethylrhodamine (TRITC)-conjugated phalloidin for 30 min to stain F-actin. Vinculin staining was accomplished by incubating the sample with mouse anti-vinculin primary antibody for 1 h, followed by incubating with FITC-conjugated goat anti-mouse IgG secondary antibody for 1 h. The visualization of nucleus was achieved by counter-staining with 4′,6-diamidino-2-phenylindole (DAPI) for 5 min. The stained cells were observed under a confocal laser scanning microscope (Zeiss LSM 510 Meta, Oberkochen, Germany) for green, red and blue fluorescence originated from vinculin, actin cytoskeleton and nucleus in stained cells. A control with 3T3 cells seeded on TCPS plates was similarly stained and imaged.

### 3.6. In Vivo Studies 

#### 3.6.1. Animal Model

All animal experiment procedures carried out in this study were approved by the Institutional Animal Care and Use committee (IACUC) of Chang Gung University. A total of forty 3-month-old New Zealand white rabbits (National Laboratory Animal Breeding and Research Center, Taipei, Taiwan) were used in this study. The rabbits were grouped into three experimental groups (treated with Tk, Tn or Tn^+^ CSNFMs), a positive control group (treated with SurgiWrap^®^), and a negative control group (untreated), with eight rabbits in each group. A flexor digitorum profundus (FDP) tendon rupture model of the hind paw similar to our previous study was used [22]. After the tendon sheaths were removed and the zone-II FDP tendons ruptured and repaired, each repair site is wrapped with an 8 × 10 mm strip of membrane (Tk, Tn or Tn^+^) for the experiment group and SurgiWrap™ for the positive control group. The negative control group was left untreated. Three weeks post-operatively, the rabbits were euthanized and the hind paws were removed at the ankle joints.

#### 3.6.2. Gross Evaluation and Biomechanical Measurements

Three weeks post-operatively, each FDP tendon is photographed for gross evaluation to determine the severity of peritendinous adhesion. For biomechanical measurements, the range of motion for the distal interphalangeal (DIP) and proximal interphalangeal (PIP) joints were measured as the flexion angle. Tendon gliding is measured as the amount of tendon excursion observed when 1 N of pulling force is exerted on the FDP tendon. Post-operative tendon adhesion is evaluated by the pull-out force (maximum force required to fully remove a tendon from its sheath). The possible effect of treatment on the normal process of tendon healing is assessed by measuring the breaking force of healed tendon (the maximum tensile force that a tendon would withstand before its breakage).

#### 3.6.3. Blood Test

To examine the possible adverse effect induced by the treatment, blood tests of the animals were conducted one week post-operatively. Complete blood count, and renal function (creatinine and uric acid) as well as liver function (alanine aminotransferase and aspartate aminotransferase) were assessed. 

#### 3.6.4. Histological Examination

The harvested samples were serially dehydrated in ethanol and the dehydrated specimens were then embedded in paraffin wax. The tissue block is shaved with a microtome knife to a thickness of 5 µm. The sections are mounted on glass slides that are heated to 70 °C and immersed in xylene for 5 min, repeating for three times before deparaffinizing the slides. The slides were then rehydrated for hematoxylin and eosin (H&E) and Masson’s trichrome staining as well as immunohistochemical (IHC) staining of F4/80 and TNF-α.

### 3.7. Statistical Analysis

The mean and standard deviation were calculated for all data. The one-way analysis of variance (ANOVA) was used for statistical analysis with SPSS 10.0 (Chicago, USA). A p-value less than 0.05 were considered statistically significant. The post-hoc analysis was carried out with Fisher’s Least Significant Difference (LSD) test. 

## 4. Conclusions

In this study, we successfully prepared functional HA-PLA/Ag NPs CSNFMs with different sheath length scale and core diameter. By controlling the release rate of HA from the core and Ag NPs from the sheath, we demonstrate the best anti-adhesion outcomes offered by large-core/thin-sheath nanofibers with embedded Ag NPs (Tn^+^) in vivo. In vitro cell experiments confirm that the Tn^+^ CSNFM can reduce attachment and prevent penetration of fibroblasts without exerting pronounced cytotoxicity in vitro. In addition, the Ag NPs-containing membranes are endowed with anti-bacterial activity by releasing Ag. In vivo studies with different CSNFMs and SurgiWrap^®^ in a rabbit flexor tendon rupture model demonstrated that as a barrier membrane, the Tn^+^ CSNFM can effectively reduce inflammation and prevent peritendinous adhesion after tendon surgery, based on gross observation, histological analysis, and functional assays.

## Figures and Tables

**Table 1:** The elemental composition from X-ray photoelectron spectroscopy (XPS) analysis.

Sample	Elemental Composition (Atomic %)			
	C	N	O	Ag
Tk	79.01	0.56	20.43	0.00
Tn	81.35	0.84	17.81	0.00
Tk^+^	78.23	0.64	21.10	0.03
Tn^+^	78.81	0.63	20.55	0.01

**Table 2:** The permeability coefficient of BSA through different CSNFMs and SurgiWrap^®^.

Sample	Tk	Tn	Tk ^+−^	Tn^+^	SurgiWrap^®^
Permability coeffecient (10^−5^ cm/s)	5.66 ± 1.05	5.89 ± 1.07	5.00 ± 0.92	5.26 ± 0.67	5.39 ± 0.81 × 10^−3^

## Data Availability

The data presented in this study are available on request from the corresponding author.
